# Pelvic shape predisposes for pelvic organ prolapse: a geometric morphometry study

**DOI:** 10.1002/uog.70101

**Published:** 2025-10-09

**Authors:** E. Stansfield, K. Phan, B. Fischer, J. O. Delancey, W. Umek

**Affiliations:** ^1^ Evolutionary Biology University of Vienna Vienna Austria; ^2^ Department of Transfusion Medicine and Cell Therapy Medical University of Vienna Vienna Austria; ^3^ Konrad Lorenz Institute for Evolution and Cognition Research Klosterneuburg Austria; ^4^ Department of Obstetrics and Gynecology University of Michigan Ann Arbor MI USA; ^5^ Department of Obstetrics and Gynaecology Medical University of Vienna Vienna Austria

**Keywords:** geometric morphometry, pelvic floor, pelvic organ prolapse, pelvic shape, risk factor

## Abstract

**Objective:**

To identify morphological features of the soft tissue and bony pelvis that could serve as predictive risk factors for the development of pelvic organ prolapse (POP) in adult women.

**Methods:**

This case–control study compared the shapes of the pelvic floor soft tissue and bony pelvis between three groups: parous women with POP (cases); parous women without symptoms of POP (controls); and nulliparous women. The primary dataset comprised 21 women around 50 years of age (mean ± SD, 50.3 ± 1.3 years), with seven participants in each group. Landmarks on the pelvis and urogenital hiatus were collected on magnetic resonance imaging scans. Pelvic shape was analyzed using geometric morphometry and principal component analysis. The findings were validated in a small secondary dataset of four parous women in their 30s, of whom two had POP and two were controls.

**Results:**

Significant differences were observed between cases, controls and nulliparae in the primary dataset when soft tissue shape and pelvic shape were analyzed together on principal component analysis. When the shape of the bony pelvis was considered alone, a significant difference was observed between cases and controls, with the former group exhibiting a mediolaterally wider pelvis with relatively short anteroposterior and craniocaudal diameters. This difference was generalizable to younger women in the secondary dataset.

**Conclusion:**

The shape of the pelvis in adult women affects their risk for postpartum POP. © 2025 The Author(s). *Ultrasound in Obstetrics & Gynecology* published by John Wiley & Sons Ltd on behalf of International Society of Ultrasound in Obstetrics and Gynecology.

## INTRODUCTION

One in 10 women (i.e. individuals assigned female at birth) over the age of 50 will experience pelvic organ prolapse (POP) in their life^1^. The number of North American women suffering from POP is expected to grow by 50% by 2050 owing to increasing life expectancy^2^. Many of the current cohort of 30‐year‐old women, who are expected to live, on average, to the age of 78 in the Global North^3^, will suffer from a pelvic floor disorder for over one‐third of their lifetime. Developing accurate risk‐prediction tools for POP should be prioritized to counsel women earlier in life about pregnancy management and delivery options.

At present, confirmed risk factors for POP include maternal age, body mass index (BMI), parity, vaginal delivery, defects of the levator ani muscle, urogenital hiatus size and neonatal birth weight^4^. Most of these risk factors are identified after childbirth, while prolapse symptoms typically develop later in life. A longitudinal study observed distinct trends in urogenital hiatus size among parous women who would go on to develop POP several years in the future^5^. However, whether there are parameters that can predict a predisposition to POP before childbirth remains to be determined.

From the point of view of preventive care, features that predict the occurrence of POP independently of soft tissue damage are important. Olafsdottir *et al*.^6^ and Pujol‐Gualdo *et al*.^7^ identified 25 genome‐wide loci that are correlated significantly with POP. Obstetricians have long considered the effects of variation in pelvic morphology on mode of delivery but, with respect to pelvic floor disorders, bony pelvic morphology has been largely ignored. Early attempts at finding an association between pelvic diameter and pelvic floor disorders yielded equivocal results^8^, ^9^, ^10^, ^11^. In 2021, Sammarco *et al*.^12^ reported a significant correlation between the pelvic cross‐sectional area at the level of the levator ani and POP. A recent study by Xu *et al*.^13^ found a link between the width of the pelvic canal and incontinence, suggesting that a narrower birth canal was associated with reduced pelvic floor dysfunction.

The pelvic floor has a complex shape that is not adequately reflected by the small number of traditional, linear measures. Our study aimed to identify anatomical risk factors for pelvic floor disorders. We investigated the degree to which the shape of the soft tissue and bony pelvis differed depending on parity and POP status. We hypothesized that there is an association between the shape of a woman's bony pelvis and her risk of POP.

## METHODS

### Patient selection

This was a case–control study of patients derived from two published studies, OPAL‐II^14^ and RUBI^15^. The original OPAL‐II study was a case–control study comprising two groups: parous women who presented with anterior‐predominant POP, defined as an anterior vaginal wall below the hymen (cases), and parous women with no symptoms and normal pelvic organ support on examination (controls). Exclusion criteria were any contraindications to undergoing magnetic resonance imaging (MRI), hip prosthesis, claustrophobia, prior surgery on the pelvic floor and other issues that would affect pelvic anatomy (e.g. genital anomaly). The RUBI study included nulliparous women of all ages who did not have any symptoms of pelvic floor disorder.

Our primary dataset comprised 21 women (seven cases, seven controls and seven nulliparae) around the age of 50 years (mean ± SD, 50.3 ± 1.3 years). The narrow age range was selected intentionally to control for well‐documented age‐related soft tissue variation^16^ and enable the detection of other smaller‐size effects that exist independently of age. No other exclusion criteria were applied to the individuals in the sample.

Subsequently, we evaluated whether the signal captured by the data for women in their 50s could be generalized to younger individuals. We did this by cross‐validating our findings based on 50‐year‐old women using a secondary dataset comprising four women in their early 30s. This secondary dataset included only two cases and two controls from the OPAL‐II study, primarily because of the low number of women with POP in this age group. All women volunteered for the studies and gave informed consent to use their data for scientific and research purposes.

Data for this study were obtained from the Michigan Pelvic Floor Research Group MRI and data repository, which contains anonymized scans and data from prior mechanistic studies at the University of Michigan, Ann Arbor, MI, USA (REP00000220). Secondary analysis of anonymized scans and data in this repository was approved by the institutional review board (HUM00194834).

### Imaging data

We collected three‐dimensional (3D) data of relevant structures from 25 T2‐weighted MRI scans of the pelvic area obtained during the OPAL‐II and RUBI studies, with a resolution of 0.78 × 0.78 × 5.00 mm. The 3D‐imaging software 3D Slicer (https://www.slicer.org/) was used to collect Cartesian coordinates of 3D landmarks placed with respect to relevant structures on the MRI scans (Figure S1).

Both soft tissue and bone were landmarked (Table S1). As the available MRI scans of the pelvic floor captured only a limited view of the true pelvis, bone landmarks were set to reflect the shape of the pelvic canal as outlined by the pubic and ischial bones, the position of the coccyx, sacrum and ischial spines and the centers of the hip joints (Figure S2). The shape of the urogenital hiatus was first captured by placing landmark points on its outline on each of the axial slices through its vertical portion. Then, a parametric surface was fitted through the existing points to approximate the original shape, and an equal number of geometrically homologous so‐called semi‐landmarks was generated within this surface, following standard geometric morphometric procedures^17^. Ultimately, the shapes of the pelvis and urogenital hiatus in each individual were described by 163 landmarks in 3D.

Data cleaning and transformation were carried out using original Python scripts and a range of libraries: NumPy, pandas, PyVista and SciPy. Further data analysis was performed in R (R Foundation for Statistical Computing, Vienna, Austria), with the help of Geomorph^18^, Morpho^19^ and RGL packages.

### Statistical analysis

Each individual's pelvis has a unique size and shape owing to biological variation. The 3D images obtained in the OPAL‐II and RUBI studies also had different orientations, i.e. inclination angles, owing to differences in the clinical imaging setting. Thus, superimposition was achieved by standardizing the 3D landmark configurations to remove variation due to size, position and orientation. After applying this method, the remaining variation was due only to shape differences. Previous work has reported differences in the size of the pelvic canal between women with and those without POP^12^. Therefore, we focused on the shape of the pelvis and urogenital hiatus to the exclusion of their size.

We used principal component analysis, an established technique for studying high‐dimensional data by reducing dimensionality, to assess shape variation in the primary dataset. This method defines a new coordinate system based on variation in the dataset. The first axis (the first principal component (PC1)) represents the direction of maximum variance in the landmark data. The second axis (PC2) represents the direction of the second largest component of variance in the sample and is orthogonal to PC1. As a result, most of the variation is summarized by the first few principal components. When applied to our 3D landmark data, the original 489 variables, comprising *x*, *y* and *z* coordinates of 163 landmarks on the bone and soft tissue, were reduced to 20 principal components. The first two components summarized most of the variation and are analyzed herein.

The Wilcoxon signed‐rank test was applied to determine if individual principal components significantly differentiated the study groups. Correlation was computed using Pearson's method, and the *P*‐value was obtained using a parametric test of the coefficient's difference from zero. Cohen's effect size was calculated for the most prominent differences between groups. This is a measure of the difference between two groups in terms of their pooled SD, which, for the purpose of interpretation, is separated into three bins: small effect (*d* ≤ 0.2), medium effect (0.2 < *d* < 0.8) and large effect (*d* ≥ 0.8).

In addition, we calculated linear measurements of the true pelvis using the set of pelvic landmarks. We used these, together with demographic/clinical characteristics of the women, to obtain discriminant functions and identify the variables that best differentiate between cases and controls.

## RESULTS

### Demographic and clinical characteristics

The primary dataset comprised seven parous women with POP (cases), seven parous women without POP (controls) and seven nulliparae. The groups were matched by age, ethnicity, education and income (Table 1). All women had a BMI above 24 kg/m^2^, and POP cases had the highest BMI. On average, parous women had experienced two pregnancies and had given birth twice. The standard mode of delivery was vaginal birth; from a total of 14 births in POP cases and 13 births in controls, only one was by Cesarean section in each group. The birth weight of the heaviest newborn did not differ between cases and controls. POP cases had a higher mean levator ani defect score and a higher frequency of urinary incontinence compared with controls. Nulliparae displayed signs of urinary incontinence at a higher frequency compared with controls but at a lower frequency compared with POP cases.

**Table 1:** Demographic and clinical characteristics of primary dataset, comprising parous women with pelvic organ prolapse (POP) (cases), parous women without POP (controls) and nulliparous women

Characteristic	Cases (*n* = 7)	Controls (*n* = 7)	Nulliparae (*n* = 7)
Age (years)	50.6 ± 0.7	50.1 ± 0.6	50.2 ± 2.0
Height (cm)	167.3 ± 3.4	168.8 ± 6.7	170.0 ± 6.0
Weight (kg)	82.0 ± 12.3	77.2 ± 13.1	76.0 ± 20.0
BMI (kg/m^2^)	29.3 ± 4.3	27.3 ± 5.7	27.0 ± 8.6
Ethnicity			
White	6 (85.7)	6/6 (100)	7 (100)
Black	1 (14.3)	0/6 (0)	0 (0)
Years of education	14.6 ± 2.3	16.1 ± 1.0	14.7 ± 1.6
Annual income (USD)	60 000 ± 10 000	50 000 ± 30 000	70 000 ± 20 000
Menopausal status			
Postmenopausal	2/6 (33.3)	2 (28.6)	4 (57.1)
Premenopausal	3/6 (50.0)	2 (28.6)	3 (42.9)
Perimenopausal	1/6 (16.7)	3 (42.9)	0 (0)
Urinary incontinence	5 (71.4)	1 (14.3)	3 (42.9)
Levator ani defect score*	2.4 ± 2.2	1.4 ± 1.6	N/A
High degree of injury unilaterally	2 (28.6)	1 (14.3)	N/A
High degree of injury bilaterally	2 (28.6)	1 (14.3)	N/A
Gravidity	2.1 ± 0.8	2.4 ± 0.9	N/A
Parity	2.0 ± 0.5	2.2 ± 0.4	N/A
Total Cesarean sections†	1/14 (7.1)	1/13 (7.7)	N/A
Birth weight of heaviest newborn (kg)	3.4 ± 0.5	3.4 ± 0.5	N/A

Data are given as mean ± SD, *n* (%) or *n*/*N* (%).

* Levator ani defects were scored from 0 to 3 on each side, with 0 indicating no damage and 3 indicating complete loss of muscle (high degree of injury); therefore, total score ranged from 0 to 6, with a score of ≥ 4 considered a major defect.

† Total number of Cesarean sections out of total number of births in cohort. BMI, body mass index; N/A, not applicable.

### Principal component analysis of landmarks on soft tissue and bone

**Figure 1:**
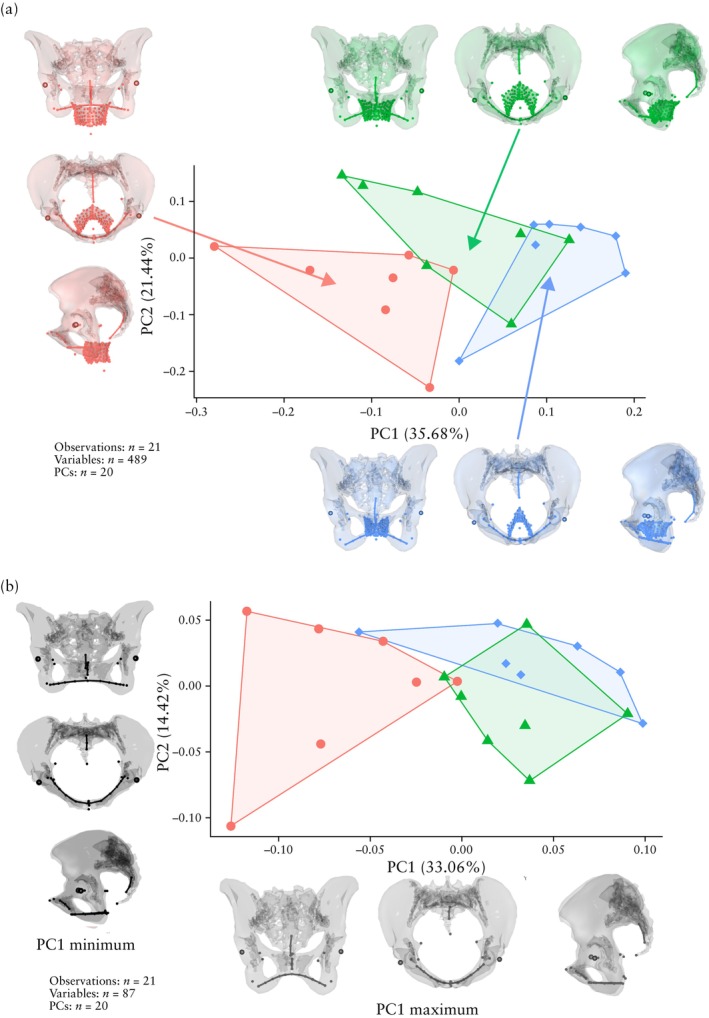
Principal component analysis of pelvic shape variation between parous women with pelvic organ prolapse (POP) (cases) (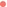), parous women without POP (controls) (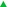) and nulliparous women (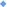) in primary dataset. (a) Three‐dimensional (3D) landmarks on bone and soft tissue; pelvic shapes reflect group means to highlight extent of intergroup differences. (b) 3D landmarks on bone only; pelvic shapes show maximum and minimum values of first principal component (PC1) to reflect direction of differences. PC2, principal component 2.

The first analysis was carried out on the entire dataset, i.e. 163 landmarks on the soft tissue and bone (489 variables). Cases, controls and nulliparae showed substantial differences in the position and shape of the levator ani (Figures 1a, S3 and S4). Differences were significant on pairwise analysis for PC1 between cases and nulliparae (*P* < 0.001) and between controls and nulliparae (*P* = 0.018), and for PC2 between cases and controls (*P* = 0.038) (Table S2). The differences are summarized by the inlays in Figure 1a, which represent the group means in the space of the first two principal components. POP cases had a wide urogenital hiatus, situated partly below the ischiopubic outline. Nulliparae displayed the opposite configuration, with a narrow urogenital hiatus sitting well above the ischial bones. Controls displayed an intermediate configuration. In addition, several differences were observed in the shape of the bony pelvis. POP cases had an anteroposteriorly shallow pelvis, which was also wider mediolaterally and shorter in the craniocaudal direction compared with the other groups.

### Principal component analysis of bone landmarks only

After excluding the soft tissue and focusing solely on bone landmarks (29 landmarks, 87 variables), substantial differences in pelvic shape between cases and controls became evident (*P* = 0.004) (Table S3, Figures S5 and S6). The pelvic shape differences are visualized at the minimum and maximum of PC1 in Figure 1b. Group differences in bony pelvis shape between cases and controls were surprisingly strong along PC1, as almost complete group separation occurred along this axis. Cases were characterized by a mediolaterally wide and anteroposteriorly shallow pelvis with a wide subpubic angle, which was also short in the craniocaudal direction. Controls had a tall pelvis with a relatively expanded anteroposterior dimension, such that the pelvic canal had a round‐to‐oval shape and a narrower subpubic angle. Pelvic shape variation in nulliparous women spanned the range of controls and cases. In the space of PC1 and PC2, nulliparae were significantly different from cases (*P* = 0.006 for PC1) but not from controls. Cohen's effect size for intergroup mean differences, as described by PC1, was 2.6.

The pelvic shape variation described by PC1 had a weak positive correlation with body height (*r* = 0.30, *P* = 0.183) and a weak negative correlation with BMI (*r* = −0.38, *P* = 0.087) (Table S4), implying that the observed differences in pelvic shape were not merely a consequence of height or BMI differences between the groups.

### Secondary dataset

#### Demographic and clinical characteristics

The secondary dataset included four individuals: two parous women with POP (cases) and two parous women without symptoms of POP (controls) (Table 2). The former were the only two individuals in the OPAL‐II study who displayed POP in their early 30s. POP cases in the secondary dataset had a lower BMI compared with cases in the primary dataset and controls in the secondary dataset. Controls in the secondary dataset were also lighter compared with their counterparts in the primary dataset. All four women had given birth at least twice. One woman with POP had delivered once by Cesarean section, but all other births were vaginal.

**Table 2:** Demographic and clinical characteristics of secondary dataset, comprising parous women with pelvic organ prolapse (POP) (cases) and parous women without POP (controls)

Characteristic	Cases (*n* = 2)	Controls (*n* = 2)
Age (years)	33.5 ± 0.5	34.0 ± 1.0
Height (cm)	174.0 ± 6.4	161.2 ± 1.7
Weight (kg)	66.9 ± 4.3	70.3 ± 3.2
BMI (kg/m^2^)	22.1 ± 0.2	27.1 ± 1.7
White ethnicity	2 (100)	2 (100)
Years of education	17.0 ± 0.0	15.9 ± 1.0
Annual income (USD)	> 60 000 ± 0	40 000 ± 20 000
Premenopausal	2 (100)	2 (100)
Urinary incontinence	2 (100)	1 (50.0)
Levator ani defect score*	3.0 ± 2.0	0.5 ± 0.5
High degree of injury unilaterally	1 (50.0)	2 (100)
High degree of injury bilaterally	1 (50.0)	0 (0)
Gravidity	2.5 ± 0.5	3.5 ± 1.5
Parity	2.0 ± 0.0	3.5 ± 1.5
Total Cesarean sections†	1/4 (25.0)	0/7 (0)
Birth weight of heaviest newborn (kg)	3.7 ± 0.3	3.3 ± 0.3

Data are given as mean ± SD, *n* (%) or *n*/*N* (%).

* Levator ani defects were scored from 0 to 3 on each side, with 0 indicating no damage and 3 indicating complete loss of muscle (high degree of injury); therefore, total score ranged from 0 to 6, with a score of ≥ 4 considered a major defect.

† Total number of Cesarean sections out of total number of births in cohort. BMI, body mass index.

#### Bone landmarks

We assessed if the detected differences in pelvic shape that were strongly associated with POP occurrence in women in their 50s could predict the presence of POP in younger individuals. The sample used here included 14 individuals from the primary dataset and four from the secondary dataset. Nulliparous women were excluded. Only bony pelvic landmarks were analyzed.

**Figure 2:**
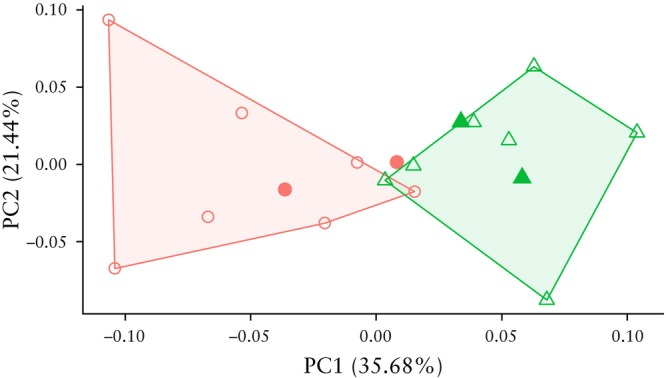
Principal component analysis of pelvic shape variation, with cases (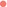) and controls (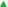) from secondary dataset mapped onto space of first (PC1) and second (PC2) principal components from primary dataset. Only three‐dimensional landmarks on bone were analyzed. 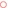, cases from primary dataset; 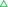, controls from primary dataset.

Principal components were extracted for the 14 individuals (seven cases and seven controls) from the primary dataset. The secondary data were standardized and then projected into the space of the first two principal components derived from the sample of women in their 50s (Figure 2). Both control individuals from the secondary dataset fitted well within the primary group of controls. One of the two additional POP cases was projected into the cloud of cases from the primary dataset. The other POP case fell close to the boundary between cases and controls.

### Linear measurements

To enable clinical application of our findings, we assessed whether any linear or angular variables measured on the pelvic bone could capture the detected 3D shape differences between cases and controls. These variables could be used in a clinical setting for POP risk assessment (Figure S7).

We performed several linear discriminant analyses to assess whether geometric variables could separate cases and controls as efficiently as well‐known POP risk factors, such as BMI, parity and levator ani injury. The coefficients obtained indicate how well each variable can differentiate between groups; the larger the absolute value of the coefficient, the more explanatory power the variable has (Table S5). The most critical geometric factors that describe the shape of the pelvis were: biacetabular width‐to‐depth ratio, inferior symphysis to coccyx depth (distance between inferior pubic symphysis and coccyx), bispinal width, bispinal‐width‐to‐pelvic‐canal‐height ratio, bispinal width‐to‐depth ratio and distance between acetabular walls.

Among the 13 tested geometric variables and patient characteristics, the SD distributions did not overlap between cases and controls for bispinal width, bispinal‐width‐to‐pelvic‐canal ratio, bispinal‐width‐to‐depth ratio, midtuberosities‐to‐superior‐pubis‐height ratio and BMI (Figure S8).

## DISCUSSION

Clinicians have long considered the effects of bony pelvic morphology on mode of delivery, but its impact on POP has not received much attention. This study provides evidence for the effect of the bony pelvis on POP. Parous women with POP had a differently shaped bony pelvis compared to parous women without symptoms of POP. Women with POP had a mediolaterally wider pelvis with relatively short anteroposterior and craniocaudal diameters. By contrast, the pelvis of parous women without POP tended to be narrower mediolaterally and deeper anteroposteriorly as well as craniocaudally.

The effect size was surprisingly large despite the small sample size, with almost complete group separation in pelvic shape observed between cases and controls even though the primary dataset comprised only 21 individuals. It is important to note that principal component analysis does not enhance group differences. This method is entirely agnostic of the group structure in the sample and cannot ‘overfit’ the data. The strong separation between groups is a consequence of the pronounced signal in the data and the 3D‐landmark‐based methodology utilized, which allowed for the analysis of more complex features compared with previous approaches based on linear dimensions.

Our finding of the relationship between the larger size of the urogenital hiatus and the presence of POP is consistent with those of previous studies^5^, ^20^. We also observed that the position of the pelvic floor muscles was different in the presence of POP: the lower boundary of the muscles fell below the ischial bones, in agreement with previously published work^21^, ^22^. However, differences in the shape of the pelvic floor soft tissue were noted not only between POP cases and the other two groups, but also between controls and nulliparae. This suggests that the shape of pelvic floor soft tissue is influenced by multiple factors. It may change after childbirth and be further affected by POP. Therefore, pelvic floor soft tissue shape – aside from the shape of the pelvic canal – does not appear to be a useful marker for the assessment of POP risk.

We have shown that the observed differences in pelvic shape could not be explained by differences in body size^23^, because pelvic shape differences were not strongly correlated with either body height or BMI. Nor can the variation in pelvic shape be explained by differing ethnic origin, as women who participated in the source studies came mainly from the same area in the USA, with White women overrepresented in all groups. The 25 women included in our primary and secondary datasets formed a homogeneous group. Nevertheless, regional variation in pelvic shape across the globe should be expected^24^, ^25^, and a broader, more geographically diverse patient sample would be required to test whether the pelvic differences identified herein are generalizable across ethnic groups.

A strength of our study was that we controlled for age by selecting individuals from a very narrow age range of around 50 years. Age is a well‐known factor associated with the occurrence of POP. We suspect that differences in pelvic shape would have been more challenging to find had our primary sample included women from puberty until postmenopause. Kolesova *et al*.^26^ and Waltenberger *et al*.^27^ have previously identified longitudinal pelvic shape changes in human females between early adulthood, childbearing age and postmenopause. In contrast, our study found that the principal components of pelvic shape from the primary sample of 50‐year‐old women successfully separated 30‐year‐old women with and those without POP based on their pelvic shape.

Our findings are consistent with those of previous publications that highlighted differences in the diameter and area of the pelvic canal in association with POP^12^, ^13^. We have shown that the pelvic canal differs between cases and controls, not only in mediolateral diameter but also in anteroposterior depth and craniocaudal height. These shape differences corroborate a study of pelvic floor biomechanics by Stansfield *et al*.^28^, which demonstrated that the anteroposteriorly elongated pelvic floor is more stable under increased intra‐abdominal pressure compared with the mediolaterally elongated one, with the latter being more prone to pelvic floor disorders.

To make our findings clinically applicable, we assessed whether specific linear measurements of the female pelvis can discriminate between women with and those without POP after delivery. The measurements we tested included ratios, widths, heights and lengths of the pelvic canal, all of which essentially describe pelvic shape. They were chosen to capture the 3D changes identified in this study: women with POP tend to have a mediolaterally wider but anteroposteriorly and craniocaudally shallower pelvis. We propose that the measures identified here, including biacetabular width‐to‐depth ratio, inferior symphysis to coccyx depth, bispinal width, bispinal‐width‐to‐pelvic‐canal‐height ratio, bispinal width‐to‐depth ratio and distance between acetabular walls, have the potential to guide the counseling of women on their personal risk of prolapse years after delivery. However, more data are required to test the predictive capacity of these measurements.

Manual pelvic measurements during a gynecological examination are no longer included in clinical guidelines. Until the 1990s, they were commonly used to predict the outcome of labor and inform the decision between Cesarean section and vaginal delivery. As part of the obstetric examination, the diagonal conjugate (inferior pubic ligament to promontory) was measured manually to predict the obstetric conjugate (retropubic eminence to sacral promontory), which represents the narrowest distance in the pelvic inlet and was inaccessible to the examiner^29^. With the introduction of imaging techniques, pelvic anatomy can be assessed with higher precision and in a less invasive manner. Clinical pelvimetry became less important when deciding on the mode of delivery^30^. However, a thorough analysis of pelvic morphology has not been used to assess the risk for gynecological conditions other than obstructed labor. Given the results of the present study, imaging and quantifying the pelvic shape may be beneficial for evaluating the risk of POP.

In conclusion, we have shown that the shape of the pelvis in parous women with POP at 50 years of age differs significantly from that in parous women of the same age who did not exhibit POP at the time of assessment. This finding builds on the existing literature on the role of pelvic canal size in determining the risk of POP. We found a large effect size in the cumulative dimension that separates the two groups of women and demonstrated its capacity to generalize to a younger age group (women in their 30s). As a result, we argue that the shape of the pelvis in adult women affects the risk of postpartum POP. However, more data are required to test the predictive capacity of linear measurements of pelvic shape.

## Supporting information


**Figure S1** Three‐dimensional landmark data collection on magnetic resonance images using 3D Slicer ( https://www.slicer.org/).
**Figure S2** Position of three‐dimensional landmarks on pelvic bone: (1) sacral intervertebral points; (2) ischial spines, right and left; (3) ischial tuberosities, right and left; (4) superior symphysis point; (5) inferior symphysis point; (6) femur head center, right and left; (7) acetabulum (wall), right and left; and (8) ischiopubic outline. Ischial tuberosities and coccyx points are not visible.
**Figure S3** Principal component analysis of landmarks on soft tissue and bone: proportion of variance described by components.
**Figure S4** Principal component analysis of landmarks on soft tissue and bone: shape differences described by first two principal components.
**Figure S5** Principal component analysis of bone landmarks only: proportion of variance described by components.
**Figure S6** Principal component analysis of bone landmarks only: shape differences described by first principal component.
**Figure S7** Linear dimensions calculated from landmark data: (1) distance between femoral head centers; (2) bispinal width; (3) distance between acetabular walls; (4) ischiopubic angle; (5) midtuberosities‐to‐superior‐pubis height; (6) coccyx‐to‐third‐sacral‐vertebra height; and (7) inferior‐symphysis‐to‐coccyx depth.
**Figure S8** Boxplots for group distributions of 12 out of 13 geometric variables and patient characteristics in discriminant function.
**Table S1** Three‐dimensional pelvic landmarks.
**Table S2** Principal component analysis of landmarks on soft tissue and bone: separation of groups tested by pairwise Wilcoxon test.
**Table S3** Principal component analysis of bone landmarks only: separation of groups tested by pairwise Wilcoxon test.
**Table S4** Correlation of first pelvic shape component (PC1) with body height and body mass index (BMI).
**Table S5** Discriminant function weights indicating capacity of geometric variables and patient characteristics to differentiate between cases and controls.

## Data Availability

The data that support the findings of this study are available on request from the corresponding author. The data are not publicly available due to privacy or ethical restrictions.
