# Supplementary material for: Pelvic shape predisposes for pelvic organ prolapse: a geometric morphometry study
Source: Ultrasound Obstet Gynecol. 2025 Oct 9;66(5):659–66. doi: 10.1002/uog.70101 (PMC12579777; doi:10.1002/uog.70101)

# Supplementary Material

##
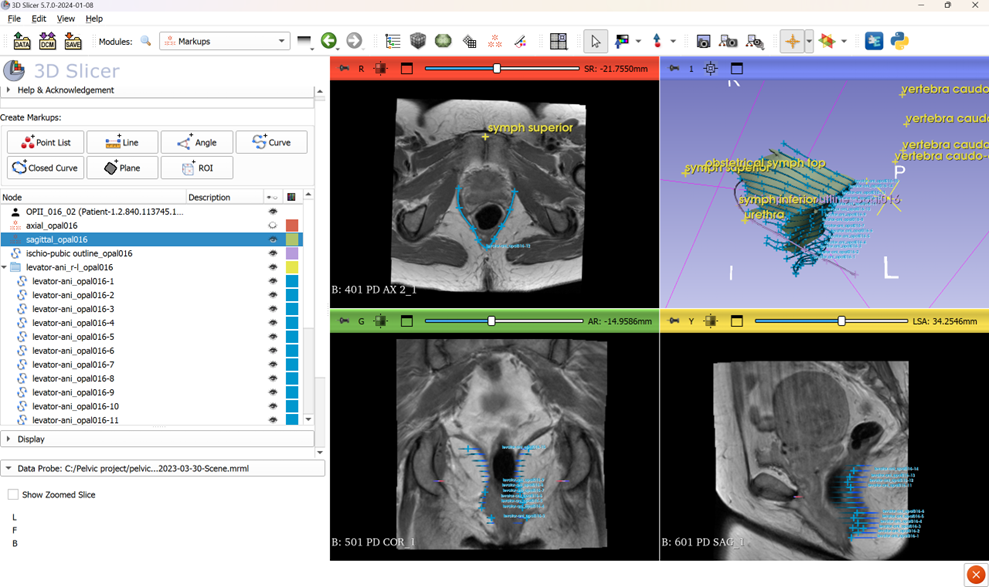
Methods

**Figure S1.** Three-dimensional landmark data collection on magnetic resonance images using 3D Slicer (https://www.slicer.org/).

**Table S1.** Three-dimensional pelvic landmarks

| Landmarks | As collected | As modified |
| --- | --- | --- |
| Levator ani outline | Points on the outer surface of the vertical part of the levator ani (where muscle is present) or levator hiatus (where muscle is absent). Nine points start and end at the insertion in the obturator fascia or pubic bone. The total number of points differs between individuals. | The points are used to fit a surface. The surface points are then re-sampled to create 102 geometrically homologous surface semilandmarks. |
| Urethra | On the sagittal slice, the lowest point of the urethra | na |
| Anus | On the sagittal slice, the lowest central point of the anal sphincter outline | na |
| Inferior symphysis point | On the sagittal slice, the lower point of the long axis of the symphysis | na |
| Superior symphysis point | On the sagittal slice, the top point of the long axis of the symphysis | na |
| Upright posture symphysis top | This is the most superior point of the pubic symphysis in the upright standing position. On the sagittal slice, it is defined as the vertical edge of the top of the symphysis, which is also the closest to the coccyx | na |
| Coccyx | On the sagittal slice, the tip of the final coccyx vertebra | na |
| Sacral intervertebral points (3-6) | On the sagittal slice, points between the coccyx and ultimate sacral vertebra and points between the sacral vertebra, the number depends on the extent of the image | Only three ultimate sacral intervertebral points |
| Femur head centre right and left | On axial slices, controlled by the femur head view on coronal slices | na |
| Acetabulum right and left | The external wall of the acetabulum on the same axial slice as the femur head centre | na |
| Ischial tuberosity on the right and left | Posterior most points of ischial tuberosities on the axial view | na |
| Ischial spine right and left | The most prominent tip of the ischial spine on the axial view | na |
| Symphysis centre on axial slices | Collected on each axial slice between inferior symphysis and obstetric symphysis points | Resampled with the help of a cubic spline to 3 curve semilandmarks |
| Ischiopubic outline | On the axial slice (perpendicular to the sagittal slice), outline ischial and pubic bones at the level of the inferior symphysis point, between the most posterior points on ischial tuberosities | na |


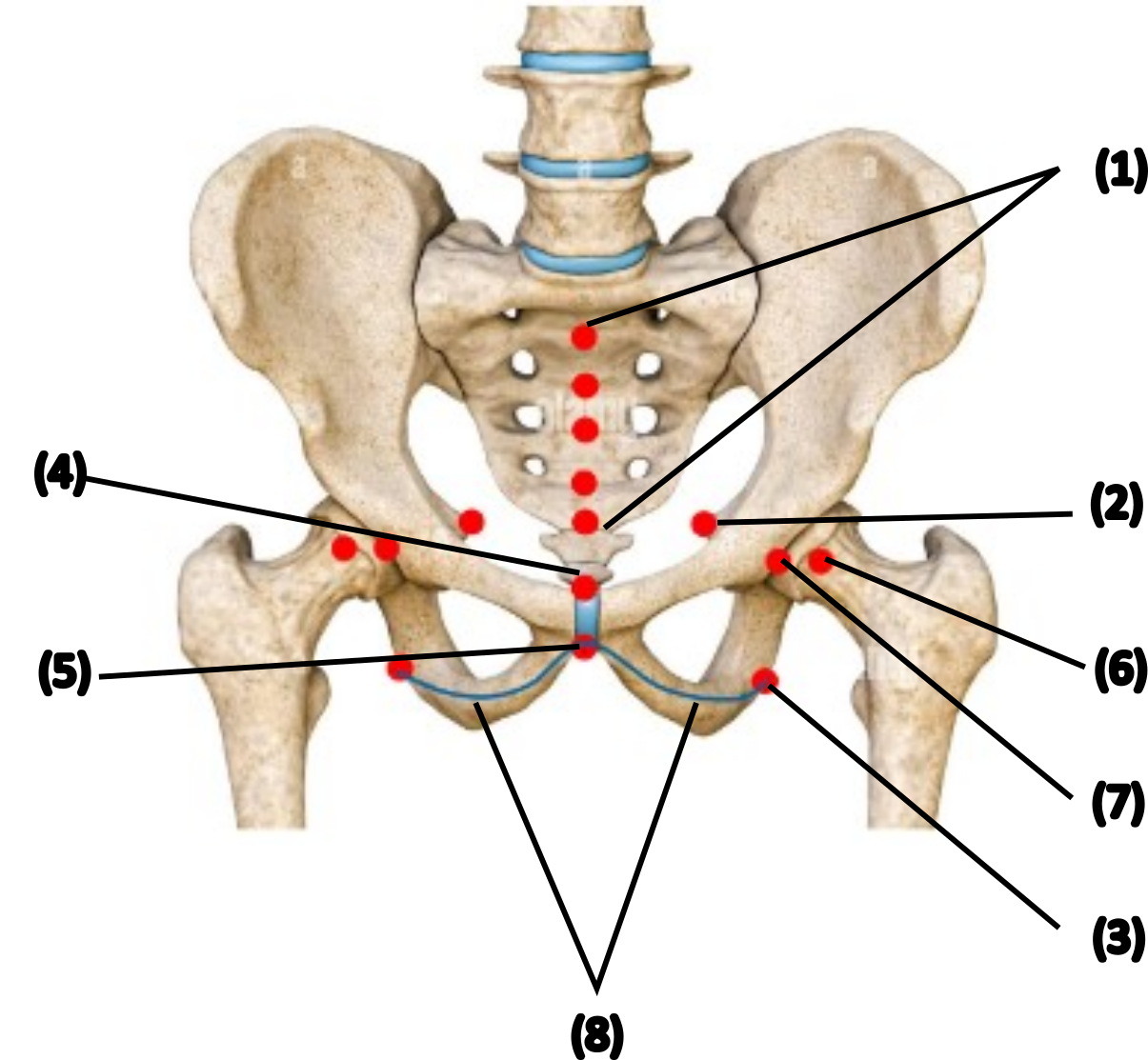


## **Figure S2.** Position of three-dimensional landmarks on pelvic bone: (1) sacral intervertebral points; (2) ischial spines, right and left; (3) ischial tuberosities, right and left; (4) superior symphysis point; (5) inferior symphysis point; (6) femur head center, right and left; (7) acetabulum (wall), right and left; and (8) ischiopubic outline. Ischial tuberosities and coccyx points are not visible.

## Results

### All data PCA

Ordination type: Principal Component Analysis

Centring by OLS mean

Orthogonal projection of OLS residuals

Number of observations: 21

Number of variables: 489

Number of vectors 20

**
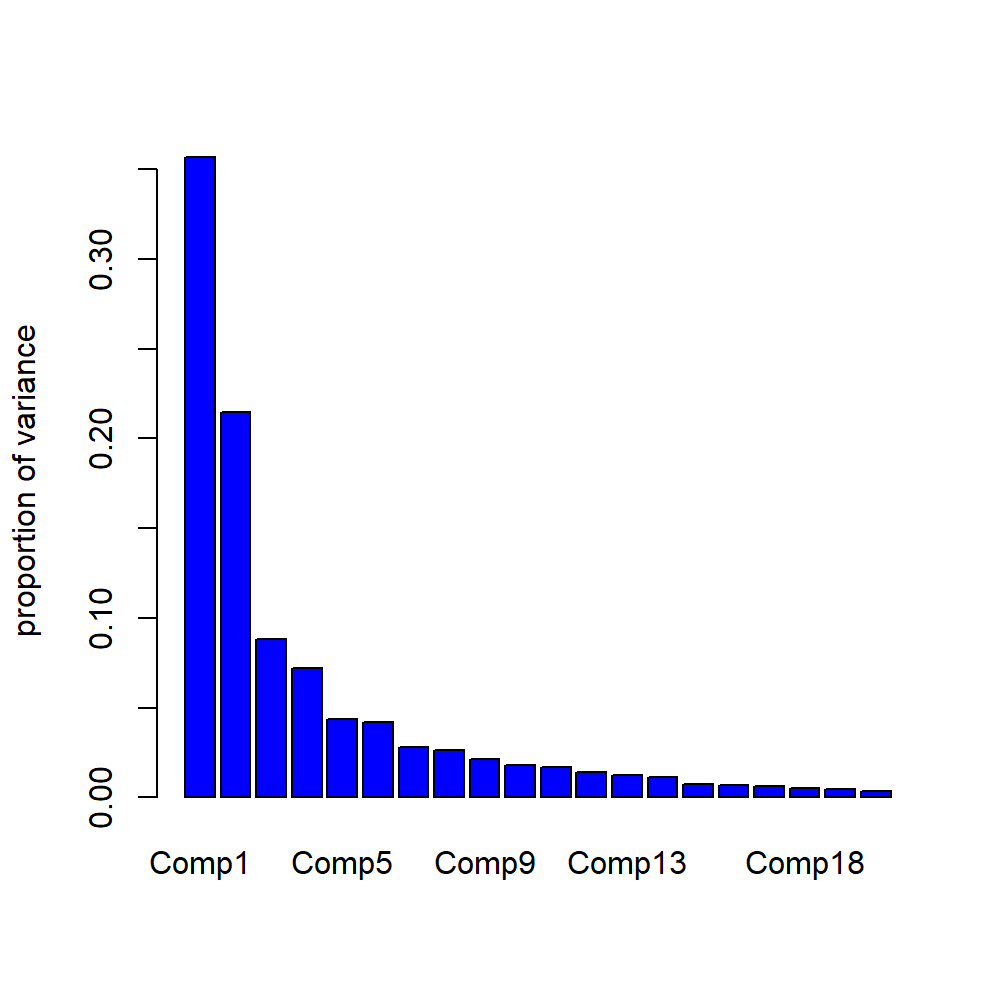
**

**Figure S3.** Principal component analysis of landmarks on soft tissue and bone: proportion of variance described by components.

**Table S2.** Principal component analysis of landmarks on soft tissue and bone: separation of groups tested by pairwise Wilcoxon test.

| Comparison | PC1 p-value | PC2 p-value |
| --- | --- | --- |
| Cases v. Controls | 0.209 | 0.038 |
| Cases v. Nullipara | < 0.001* | 0.073 |
| Controls v. Nullipara | 0.018 | 0.456 |

*Significant at 0.01 level

|  | **PC1** |
| --- | --- |
| **Min**  **Max** | 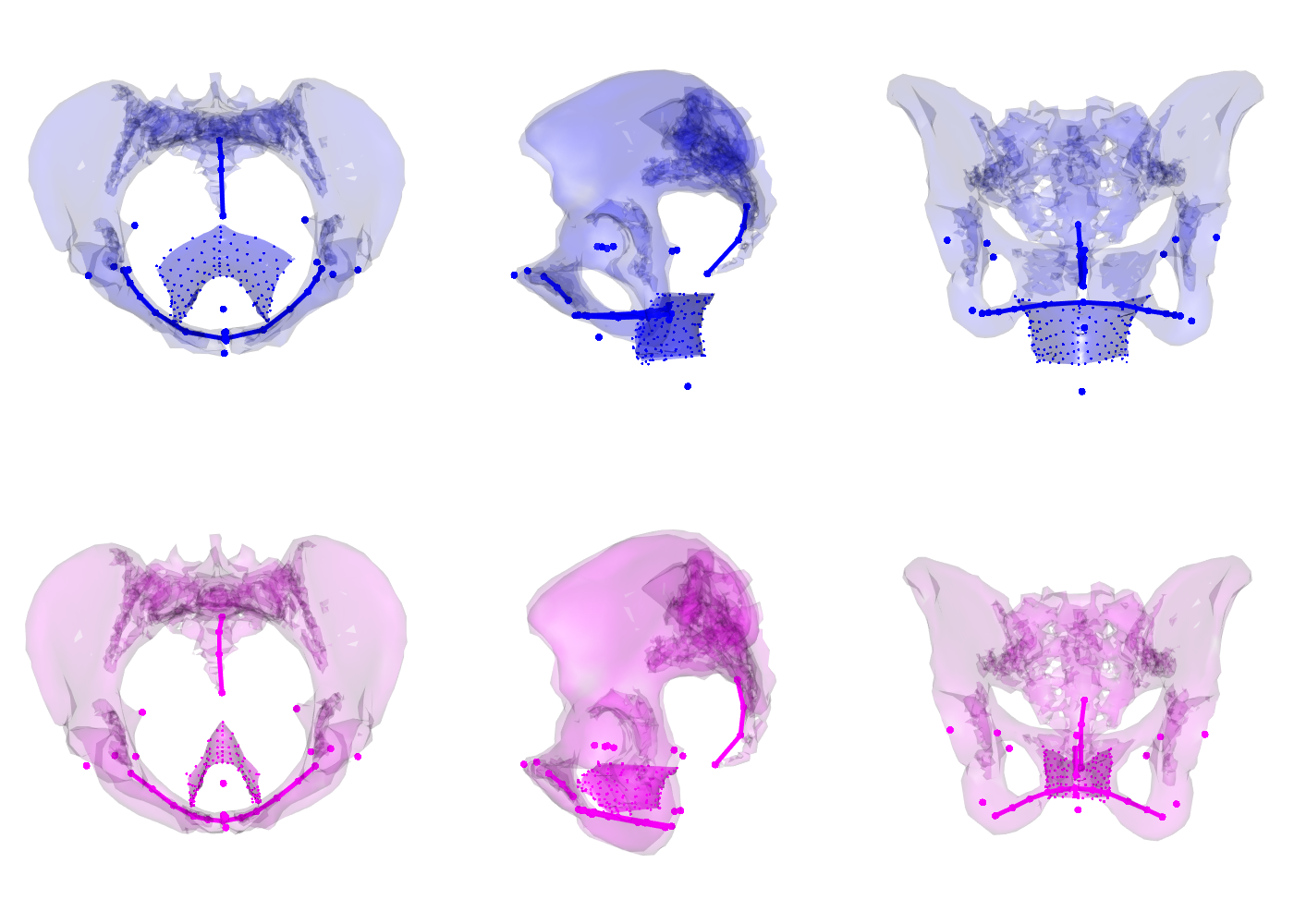 |

|  | **PC2** |
| --- | --- |
| **Min**  **Max** | 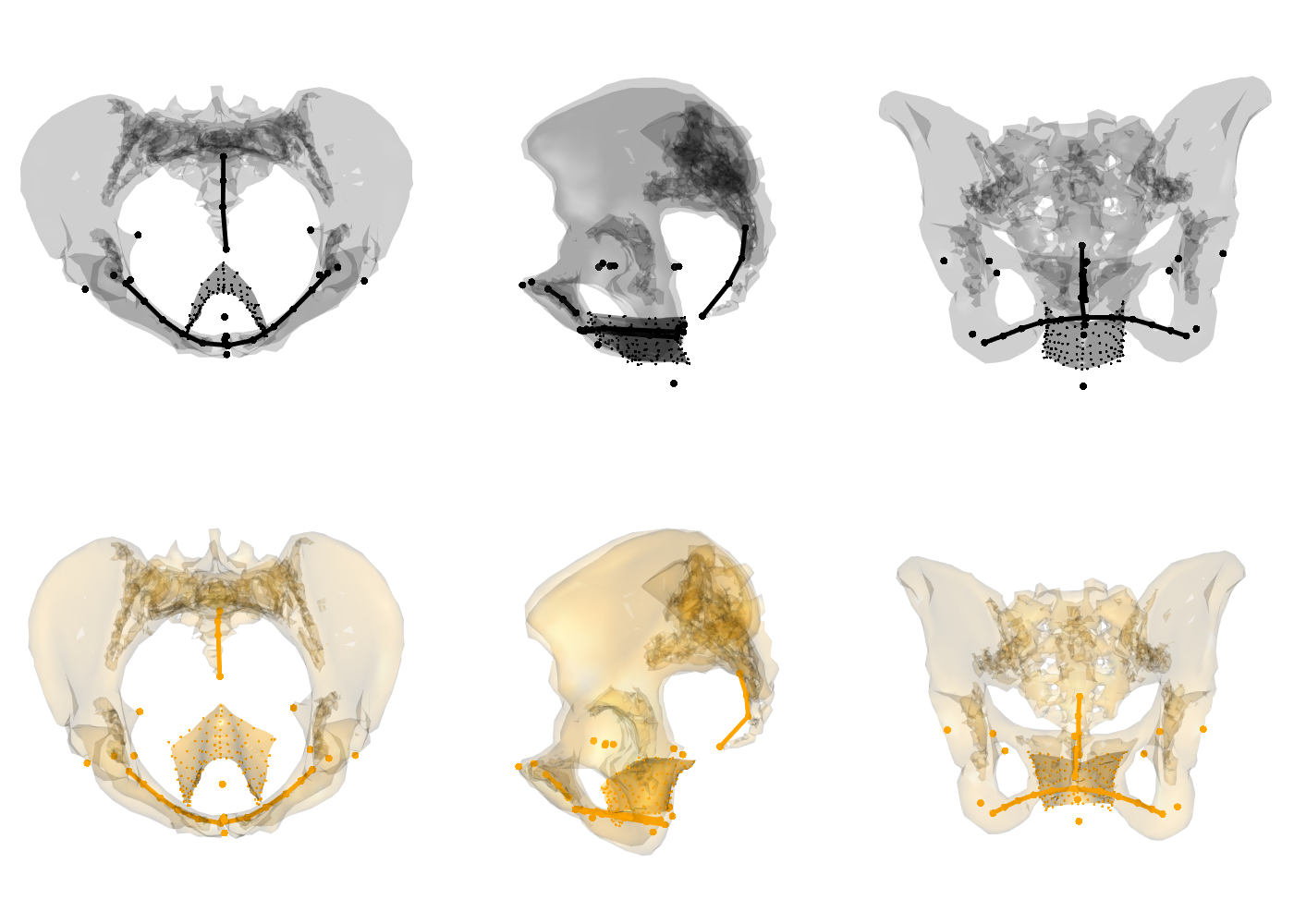 |

**Figure S4**. Principal component analysis of landmarks on soft tissue and bone: shape differences described by first two principal components.

### Bone only PCA

Ordination type: Principal Component Analysis

Centring by OLS mean

Orthogonal projection of OLS residuals

Number of observations: 21

Number of variables: 87

Number of vectors 20


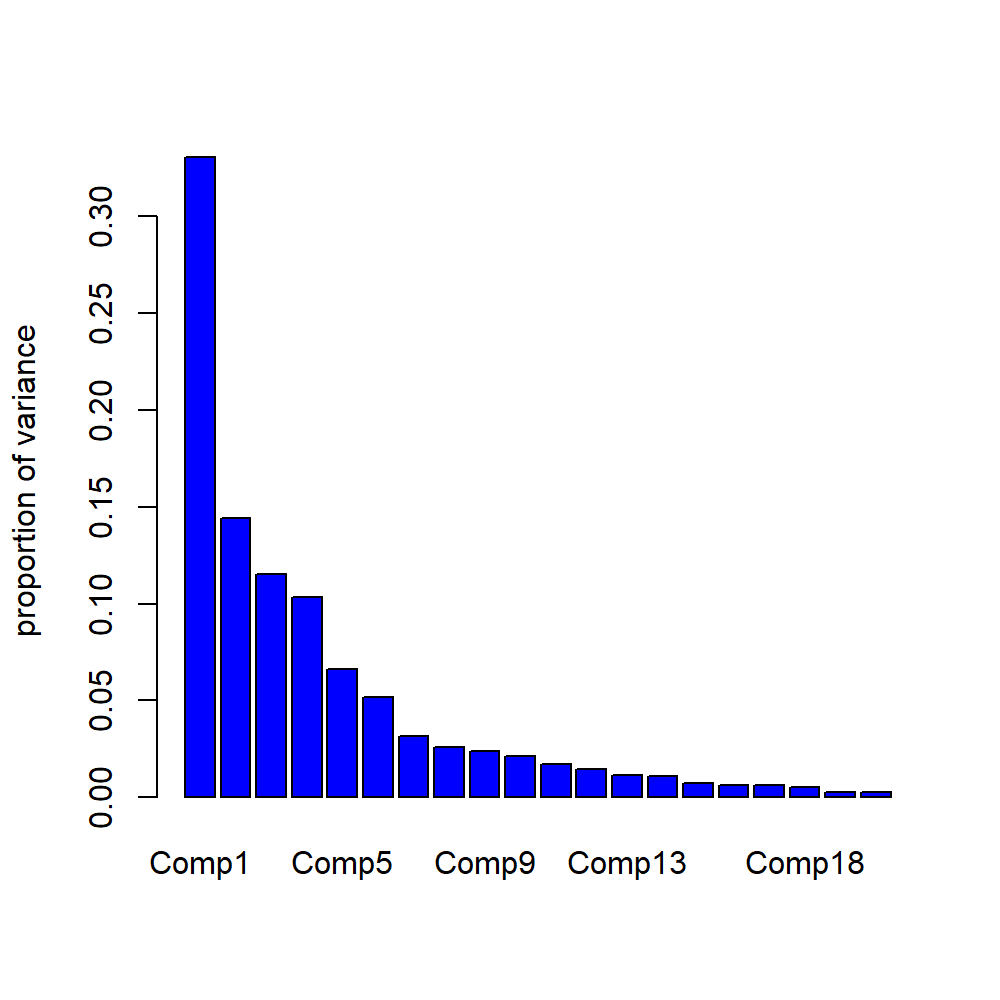


**Figure S5.** Principal component analysis of bone landmarks only: proportion of variance described by components.

**Table S3**. Principal component analysis of bone landmarks only: separation of groups tested by pairwise Wilcoxon test.

| Comparison | PC1 p-values | PC2 p-values |
| --- | --- | --- |
| Cases v. Controls | 0.004* | 0.620 |
| Cases v. Nullipara | 0.006* | 0.620 |
| Controls v. Nullipara | 0.710 | 0.160 |

*Significant at 0.01 level

| **PC1** | |
| --- | --- |
| **Min** | 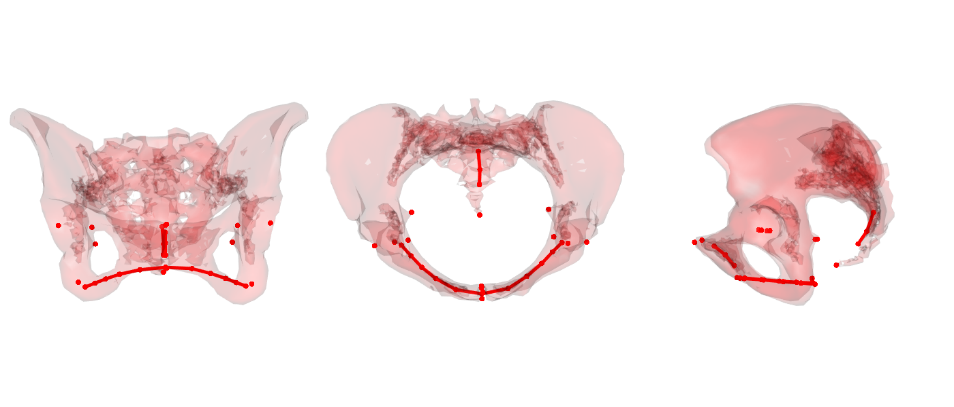 |
| **Max** | 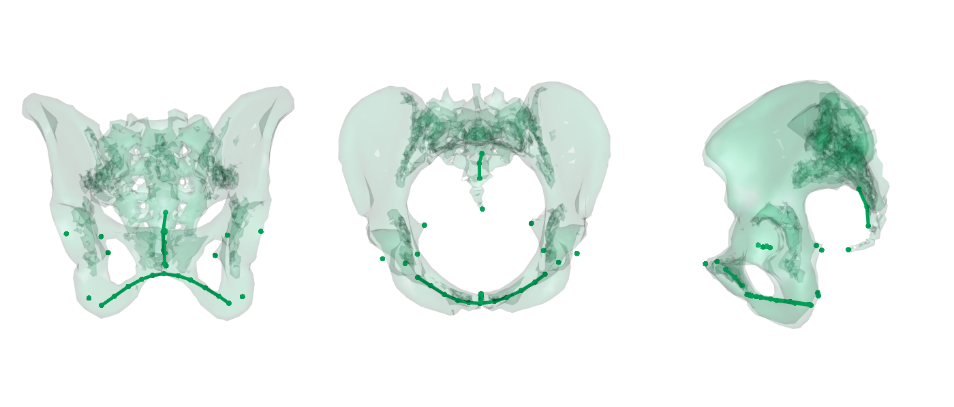 |

**Figure S6.** Principal component analysis of bone landmarks only: shape differences described by first principal component.

### Pearson’s correlation

**Table S4**. Correlation of first pelvic shape component (PC1) with body height and body mass index (BMI).

|  | r | df | p-value |
| --- | --- | --- | --- |
| PC1 v. body height | 0.30 | 19 | 0.183 |
| PC1 v. BMI | -0.38 | 19 | 0.087 |


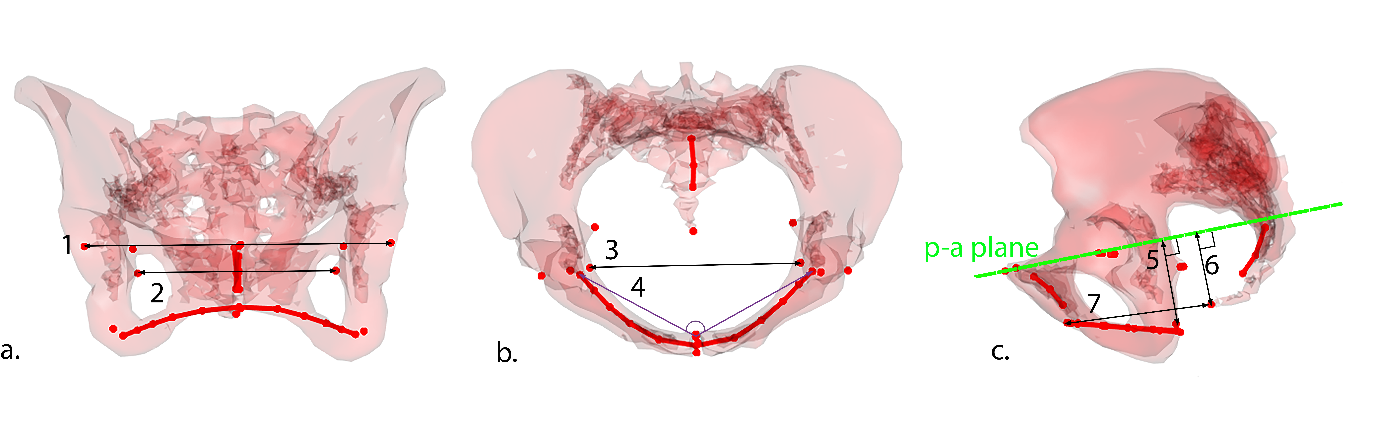
**Figure S7.** Linear dimensions calculated from landmark data: (1) distance between femoral head centers; (2) bispinal width; (3) distance between acetabular walls; (4) ischiopubic angle; (5) midtuberosities to superior pubis height; (6) coccyx to third sacral vertebra height; and (7) inferior symphysis to coccyx depth. p–a plane indicates superior-pubis-to-acetabular-center plane.

**Table S5**. Discriminant function weights indicating capacity of geometric variables and patient characteristics to differentiate between cases and controls.

| Name of measurement | Coefficients |
| --- | --- |
| Bi-acetabular width to depth ratio | -40.47 |
| Inf. symph. to coccyx depth | -21.14 |
| Bi-spinal width | -18.00 |
| Bi-spinal width to pelvic canal height ratio | -16.63 |
| Mid-tuberosities to superior pubis height | -12.26 |
| Births | -7.45 |
| BMI | -5.43 |
| Distance between femoral head centers | -5.41 |
| Ischiopubic angle | -0.20 |
| LA defect score | 2.56 |
| Coccyx to 3rd Sacral vertebra height | 12.64 |
| Distance between acetabular walls | 15.92 |
| Bi-spinal width to depth ratio | 16.28 |

**Figure S8.** Boxplots for group distributions of 12 out of 13 geometric variables and patient characteristics in discriminant function.


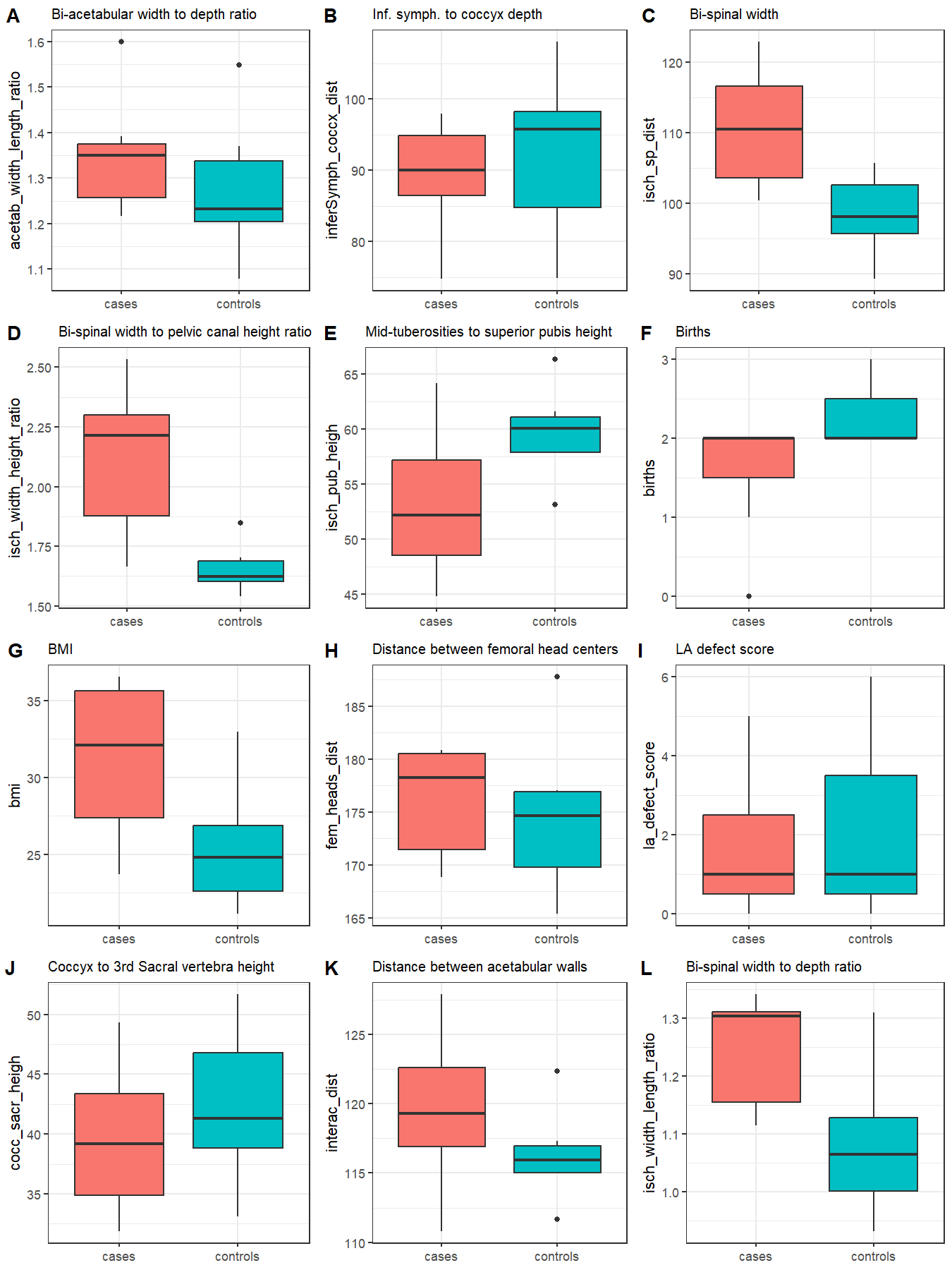

Supplement: Supplementary file 1 — Figure S1 Three‐dimensional landmark data collection on magnetic resonance images using 3D Slicer ( https://www.slicer.org/). Figure S2 Position of three‐dimensional landmarks on pelvic bone: (1) sacral intervertebral points; (2) ischial spines, right and left; (3) ischial tuberosities, right and left; (4) superior symphysis point; (5) inferior symphysis point; (6) femur head center, right and left; (7) acetabulum (wall), right and left; and (8) ischiopubic outline. Ischial tuberosities and coccyx points are not visible. Figure S3 Principal component analysis of landmarks on soft tissue and bone: proportion of variance described by components. Figure S4 Principal component analysis of landmarks on soft tissue and bone: shape differences described by first two principal components. Figure S5 Principal component analysis of bone landmarks only: proportion of variance described by components. Figure S6 Principal component analysis of bone landmarks only: shape differences described by first principal component. Figure S7 Linear dimensions calculated from landmark data: (1) distance between femoral head centers; (2) bispinal width; (3) distance between acetabular walls; (4) ischiopubic angle; (5) midtuberosities‐to‐superior‐pubis height; (6) coccyx‐to‐third‐sacral‐vertebra height; and (7) inferior‐symphysis‐to‐coccyx depth. Figure S8 Boxplots for group distributions of 12 out of 13 geometric variables and patient characteristics in discriminant function. Table S1 Three‐dimensional pelvic landmarks. Table S2 Principal component analysis of landmarks on soft tissue and bone: separation of groups tested by pairwise Wilcoxon test. Table S3 Principal component analysis of bone landmarks only: separation of groups tested by pairwise Wilcoxon test. Table S4 Correlation of first pelvic shape component (PC1) with body height and body mass index (BMI). Table S5 Discriminant function weights indicating capacity of geometric variables and patient characteristics to differentiate be [file UOG-66-659-s001.docx]
